# A lowered threshold to partnerships: a mixed methods process evaluation of participants’ experiences of a person-centred eHealth intervention

**DOI:** 10.1186/s12913-023-10190-7

**Published:** 2023-11-02

**Authors:** Matilda Cederberg, Emmelie Barenfeld, Lilas Ali, Inger Ekman, Anneli Goulding, Andreas Fors

**Affiliations:** 1 Institute of Health and Care Sciences, Sahlgrenska Academy, University of Gothenburg, Box 457, Gothenburg, 405 30 Sweden; 2 Centre for Person-Centred Care (GPCC), University of Gothenburg, Gothenburg, Sweden; 3 Psychosis Clinic, Region Västra Götaland, Sahlgrenska University Hospital, Gothenburg, Sweden; 4 Institute of Neuroscience and Physiology, Section of Health and Rehabilitation, Occupational therapy, Sahlgrenska Academy, University of Gothenburg, Gothenburg, Sweden; 5 Department of Psychiatry, Sahlgrenska University Hospital, Gothenburg, Sweden; 6 Department of Internal Medicine and Geriatrics, Sahlgrenska University Hospital Östra, Gothenburg, Sweden; 7 Department of Psychology, University of Gothenburg, Gothenburg, Sweden; 8 Education, Development and Innovation, Region Västra Götaland, Primary Health Care, Research, Gothenburg, Sweden

**Keywords:** Person-centred care, eHealth, Process evaluation, Mixed methods

## Abstract

**Background:**

In order to understand pathways of complex interventions, the Medical Research Council has suggested that process evaluations should be conducted alongside randomised controlled trials (RCTs). This paper presents a mixed methods process evaluation of a complex, person-centred eHealth intervention for persons on sick leave with common mental disorders.

**Aim:**

The aim of the study was to explore participants’ experiences of a person-centred eHealth intervention and illuminate meaningful activities and processes.

**Methods:**

Participants were recruited from the intervention arm of an RCT (n = 102). Questionnaires on perceived meaningfulness of the overall intervention and intervention activities were sent to participants on two occasions, after 3 and 6 months, and semi-structured interviews were conducted with a purposeful sample of 15 participants in the intervention group. Questionnaire data were analysed using descriptive statistics, and interview data were analysed using qualitative content analysis. The quantitative and qualitative data strands were integrated at interpretation.

**Results:**

At both follow-ups, a majority of participants reported that the intervention was fully or partly meaningful and that the most meaningful activity was the phone calls with health care professionals working in the intervention. In the qualitative analysis, three categories describing participants’ experiences of the intervention were formed: *Acknowledgment in a disconcerting situation*, *Finding ways forward* and *Unmet expectations*. A synthesis of quantitative and qualitative findings resulted in the overarching theme of meaningfulness as constituted by *a lowered threshold to partnerships: support within reach, when needed*.

**Conclusion:**

Experiences of meaningfulness of the intervention were constituted by a lowered threshold to forming care partnerships, in which support was within reach, when needed. If the content of the intervention was not in accordance with individuals’ needs or expectations, access alone did not suffice to constitute meaningfulness.

**Trial registration:**

ClinicalTrials.gov; NCT03404583; 19/01/2018.

**Supplementary Information:**

The online version contains supplementary material available at 10.1186/s12913-023-10190-7.

## Background

In Sweden and other high-income countries, the past decades have shown an increase in sick leave related to common mental disorders (CMDs) such as depression, anxiety syndromes and stress-related mental illness [1–3]. Due to their high prevalence in a general population, primary mental health care, where the majority of patients with these conditions seek help [4], has been struggling to allocate the necessary resources to give patients timely and effective care [5]. Developing eHealth alternatives has been one way of improving access to care, for example, in the form of digitalised self-guided treatments, telepsychology or as part of stepped-care programmes [6, 7]. The use of mental health apps has also demonstrated the potential to improve health outcomes for persons with depression, anxiety disorder or substance abuse, and to improve treatment availability [8]. The majority of evaluated eHealth services for persons with CMDs build on cognitive behavioural therapy (CBT) and primarily focus on symptom improvement rather than outcomes related to sick leave, such as return to work [9–11]. By building on the premise of enabling patients to manage more of their condition on their own, it has been argued that eHealth services are also resource-effective [10, 12].

Person-centred care (PCC) is an approach to health care advocating that care should be co-created in partnerships between healthcare professionals and patients, and if warranted, these partnerships should extend to include other persons of relevance to the patients’ care process, such as other professional contacts, family members and friends [13, 14]. Partnerships are characterised by sharing of information and decision making, trust and mutuality. They are based on interpersonal and communicative processes in which patients’ experiences are essential [15]. In order for partnerships to be formed, patients need to be included in their care process and given the necessary space and means to participate [16]. This conviction is based in the ontological assumption that humans possess resources and that treating patients as persons entails recognising both the resourcefulness and vulnerability of each patient [17–19]. A defining feature of person-centred care is that the goal of health care interventions is not necessarily directed at managing the illness, but at understanding what constitutes a meaningful life for the patient and offer support in that direction [20]. This focus on a meaningful life is congruent with recovery-oriented initiatives in mental health care, which defines recovery as a deeply personal, individual process towards living a fulfilling and meaningful life even if there are limitations caused by illness [21].

### PROMISE: a person-centred intervention for patients on sick leave with CMDs

A research project (PROMISE) was launched in 2018 in an eHealth setting with the aim of operationalising the ethics of PCC in an intervention for persons on sick leave with CMDs [22, 23]. The intention was to evaluate whether the person-centred eHealth intervention offered in addition to usual care at the patients’ primary care centres could support patients in their sick leave process and influence self-efficacy by identifying and mobilising personal and social resources and support. Support can be conceptualised as an individual’s perception that supportive resources, such as others providing information, services or emotional reassurance, are available if needed [24].

In order to better understand the pathways of intervention programmes, especially when they entail levels of complexity, the Medical Research Council (MRC) has suggested the need to conduct process evaluations alongside effect studies [25]. Process evaluations are valuable when seeking to understand whether an intervention is working as expected and to explore potentially unexpected mechanisms, and to capture experiences of the intervention [25, 26]. A few studies have reported on patients’ experiences of eHealth interventions for CMDs. For example, in a process evaluation of a web-based blended intervention for employees on sick leave with CMDs, patients reported overall satisfaction but needed more support from health care professionals (HCPs) [27]. A meta-synthesis on participants’ experiences of digital health interventions also found that personal support from HCPs was highly valued and seen as a component influencing the intervention’s overall successfulness, as it enabled personalisation of care [28]. Similar results were found in a study on experiences of an internet mediated CBT intervention for depression [29]. To our knowledge, no one has yet explored experiences of interventions for patients with CMDs grounded in person-centred ethics. In the PROMISE intervention, the anticipated core component was the partnership, whose potential as support lies in how it is experienced by the persons involved, not in the delivery of a certain procedure [14]. Therefore, the aim of the present study was to explore participants’ experiences of a person-centred eHealth intervention and illuminate meaningful activities and processes.

## Methods

### Study setting: the PROMISE intervention

The PROMISE project recruited patients on sick leave due to CMDs, including depression, anxiety syndromes or stress-related mental illness (adjustment disorder, acute stress reaction or exhaustion disorder) from nine primary health care centres in a larger city in Sweden. To be included, sick leave had to be issued by a physician and the duration of the sick leave should not have exceeded 30 days at the time of inclusion. The recruitment, randomisation and intervention processes were managed remotely from a research setting separate from the primary health care centres by dedicated health care professionals (HCPs). The final study sample included 209 participants (107 in the control group and 102 in the intervention group). Further details on the project and the RCT have been published elsewhere [22]. Participants in both groups received usual care at their primary health care centres. Usual care commonly involves meetings with a physician, and decisions on treatments and sick leave are based on individual assessment and evidence-based guidelines. This process differs somewhat for the different conditions within the CMD spectrum but often include advice on self-care, medication, CBT and meetings with a physiotherapist, rehab coordinator, occupational therapist or group treatments [30, 31]. For participants in the intervention group, phone support from HCPs were offered on top of usual care during a period of 6 months, as well as unlimited access to a web-based platform. The intention of using a remote format for support was to enable a PCC process without requiring face-to-face meetings. See Table 1 for an overview of intervention content.

**Table 1:** Overview of intervention activities and processes

Phone support	Web-based platform
Narrative dialogues forming a health plan containing personal goals and identifying resources and needs for support Agreement on documentation and follow-up Flexible duration and frequency Scheduled according to individual agreement	Read and write health plans Daily ratings of well-being, sleep, energy and concentration Graphic visualisations of weekly ratings Keep private notes Chat with HCPs Contact details to HCPs Access to information on CMDs and advice for managing stress Invite relatives, HCPs or workplace representatives to the platform Manage accessibility (delete invited person, manage what content each invitee can access on the platform page)

HCPs = Health care professionals

CMDs = Common mental disorders

HCPs from different disciplines (nursing, psychology, physiotherapy and occupational therapy) with various experiences of working with CMDs and PCC conducted the intervention. They took part in a half-day education session on symptoms, treatment and care for CMDs led by psychologists and physicians, were introduced to the philosophical underpinnings of PCC by scholars in the area, and participated regularly in meetings where they together with each other and experts in PCC could discuss and practise person-centred communication.

During the scheduled phone conversations, HCPs encouraged the patients to narrate how they experienced their situation. With their experiences as a departure point, they discussed patient’s goals and a plan to reach them, and the HCPs were mindful of identifying personal and social resources of value in each patient’s recovery process. This agreement was documented in the form of a personal health plan, which was uploaded to the web-based platform and served as a personal plan guiding the subsequent intervention process. The conversations used a narrative approach in that the patients were encouraged to narrate their experiences related to their illness and sick leave period and collaborate actively in their care by providing the contextual information central to the health plan and tailored support. After the initial phone conversation, follow-up calls were scheduled in agreement.

The patients could access the platform using any device with an internet connection and a web browser. On the platform, patients could make daily ratings on symptoms and well-being, monitor their symptoms and keep private notes. They could seek information on their condition through links to other web pages. The platform was also intended to facilitate communication and sharing of information between the patient and the intervention HCPs as well as the patient’s extended network. Patients could engage their supportive network, such as other health care contacts, family members or workplace representatives, by inviting them to the platform in order to keep them informed. Patients could manage how much of the content each invitee could view, and they could allow or remove access independently.

### Data collection

The present study builds on questionnaire data and semi-structured interviews. To explore participants’ experiences of the intervention, a concurrent quantitative + qualitative mixed methods design was used, with quantitative and qualitative strands integrated at interpretation [32, 33].

Participants were recruited from the intervention arm of the RCT study (n = 102). Data were collected synchronously and consecutively. Quantitative data were gathered at 3 and 6 months after inclusion using questionnaires. The questionnaires contained three items on perceived meaningfulness of the support and intervention content, previously published by Barenfeld et al. [34]. In the questionnaire, meaningfulness is conceptualised as activities and processes perceived as meaningful in relation to a personal goal.

- Participants were asked to rate whether they found the intervention overall to be a meaningful support on a 5-point Likert scale with the following answer options: fully agree, partly agree, partly disagree, fully disagree, do not know *(item 1).*
- They were then presented with an 11-item list of the intervention’s content (Table 3) and asked to mark which, if any, of the content they found meaningful (*item 2*).
- An open-ended question on which, if any, content they found most meaningful (*item 3*).

Qualitative interviews were used for in-depth exploration of experiences [25, 35]. Semi-structured interviews were conducted with participants in the intervention arm of the RCT, recruited consecutively throughout the study period upon individual completion of the 6-month phase of active intervention. Eligible participants were contacted via phone by the first author. During the period of recruitment, 14 participants did not respond and 2 declined to participate; 17 agreed to participate, but at the time of the interview, 2 did not respond. A purposeful sampling procedure was conducted to ensure heterogeneity in gender, age, diagnosis and overall positive and negative experiences of the intervention, assessed through dichotomisation of item 1 in the post-intervention questionnaire. Responding that the intervention was fully or partly meaningful was considered an overall positive experience, whereas fully or partly disagreeing or not knowing were considered an overall negative experience. In the final sample of 15 participants, 4 had negative experiences of the intervention (fully, partly or did not know), and 11 had positive experiences (fully or partly). Of the 15 participants, 4 were men and 11 were women, and they were between 29 and 59 years of age. For further details, see Table 2.

**Table 2:** Participants’ characteristics and intervention use

	Intervention group (n = 102)	Interview participants (n = 15)
GENDER n, (%)		
Women	82 (80.4)	11 (73.3)
Men	20 (19.6)	4 (26.7)
AGE		
Mean years	42.3	38.6
Median (range)	41 (21–66)	37 (29– 59)
DIAGNOSIS (ICD-10) n, (%)		
Stress (F43)	65 (63.7)	10 (66.7)
Depression (F32,33)	21 (20.6)	4 (26.7)
Anxiety (F41)	16 (15.7)	1 (6.7)
EDUCATION LEVEL n, (%)		
Compulsory	6 (5.9)	1 (6.7)
Secondary school	21 (20.6)	3 (20.0)
Vocational school	15 (14.7)	1 (6.7)
University	59 (57.8)	10 (66.7)
USE OF PHONE CALLS		
Number of phone calls, median (range)	4 (0–9)	5 (4–7)
Length of phone calls in minutes, mean (SD)	32.6 (10.3)	25.7 (5.7)
USE OF PLATFORM FUNCTION		
Number of self-ratings, median (range)	3 (0-170)	7 (0–108)
Invited family or professional contacts, median (range)	0 (0–4)	0 (0–4)
Written messages to HCPs, median (range)	0 (0–22)	0.5 (0–10)

ICD-10 = International Statistical Classification of Diseases and Related Health Problems.

HCP = Health Care Professional.

The first author conducted the interviews, each lasting between 25 and 64 min. Participants were invited to choose whether to be interviewed in person or via telephone. All but one participant chose the telephone option. A semi-structured interview guide was developed assessing experiences of the intervention as a whole as well as its content and composition (supplementary material). All interviews started with asking the participants to describe how they were doing at the time they got access to the intervention and how they felt about what the intervention offered. Thereafter, they were asked to describe what it was like for them to take part in the intervention, how they experienced the phone calls and the platform and their overall process of recovery.

### Data analysis

For the questionnaire data, descriptive statistics were used to illustrate responses on the intervention’s meaningfulness after 3 and 6 months. The descriptive statistics were calculated by the first author using SPSS. Data on most meaningful content were gathered either from participants who had listed only one activity in the question containing multiple options (item 2) or through participants’ answers to the open-ended question on which activity they found most meaningful (item 3). As this item was open-ended, after all answers had been read through, they were sorted into one of the following five categories: phone communication; rating & monitoring symptoms; health plan; using links; or two or more activities. The ‘two or more activities’ category comprised answers where participants explicitly stated that both activity a and activity b were most meaningful, or answers where participants indicated that a combination of activities was most meaningful, for example, talking with the HCPs and making a health plan.

The interviews were analysed using qualitative content analysis according to Graneheim and Lundman [36, 37]. An inductive approach was chosen to explore participants’ experiences of the intervention support. To begin, the first author read through all the transcripts several times to get an initial grasp of the content. Meaning units were then identified on a manifest level throughout all transcripts using NVivo and sorted into content areas. Thereafter, the meaning units were condensed, and the condensations were abstracted into codes. All codes were then compared in terms of differences and similarities and sorted into main categories and subcategories [36, 37]. The first author conducted the initial categorisation, which was developed in collaboration with the last author. The categories were then discussed several times together with all authors, including excerpts from the transcripts to ensure transparency of and closeness to data. In the presentation of findings, quotes from the interviews are used to express the participants’ voices and to illustrate the categories. Each interview participant was allocated a number between 1 and 15 to which they are referred to in the result section.

The data from the questionnaires and the data from the interviews were analysed separately and the results were integrated at interpretation level [32, 33]. The integration of findings was performed in a last step of the analytic procedure in a synthesis of both quantitative and qualitative findings. The synthesis was performed as an interpretation of the material as a whole in the form of an overarching theme on meaningful activities and processes in the intervention and what enabled or blocked participants’ experience of the intervention as meaningful.

## Results

### Meaningfulness of the intervention

Altogether, 84 of the 102 participants in the intervention group responded to the questionnaire at the 3-month follow-up (n = 18 missing), and 81 responded at the 6-month follow-up (n = 21 missing).

**Table 3:** Meaningfulness of intervention overall and intervention activities after 3 and 6 months

	3 months (n = 84)	6 months (n = 81)
OVERALL MEANINGFULNESS (%)^a^		
Fully agree	34 (41.0)	27 (33.3)
Partly agree	36 (43.4)	36 (44.4)
Partly disagree	7 (8.4)	7 (8.6)
Fully disagree	3 (3.6)	5 (6.2)
Do not know	3 (3.6)	6 (7.4)
MEANINGFUL ACTIVITY (%)^b^		
Scheduled phone communication	73 (86.9)	65 (80.2)
Rating symptoms	29 (34.5)	21 (25.9)
Monitoring symptoms over time	19 (22.6)	17 (21.0)
Inviting significant others	5 (6.0)	2 (2.5)
Contacting intervention staff through the platform	27 (32.1)	29 (35.8)
Contacting intervention staff by phone	18 (21.4)	19 (23.5)
Reading health plan	42 (50.0)	31 (38.3)
Writing health plan	15 (17.9)	9 (11.1)
Accessing links to condition-specific information	17 (20.2)	22 (27.2)
Other	2 (2.4)	1 (1.2)
Nothing	5 (6.0)	8 (9.9)
MOST MEANINGFUL ACTIVITY (%)^c^		
Phone communication	46 (70.8)	39 (67.2)
Rating & monitoring symptoms	10 (15.4)	7 (12.1)
Reading or writing health plan	3 (4.6)	1 (1.7)
Accessing links to condition-specific information	1 (1.5)	2 (3.4)
Two or more activities	5 (7.7)	9 (15.5)

^a^ One missing answer at the 3-month follow-up

^b^ Possible to choose more than one activity

^c^ n = 65 responses at the 3-month follow-up and n = 58 responses at the 6-month follow-up.

At 3 months, 41% (n = 34) responded that the intervention overall was fully meaningful, and 43.4% (n = 36) responded that it was partly meaningful. Scheduled phone communication was reported as meaningful by 86.9% (n = 73), and 70.8% (n = 46) reported the phone communication to be the most meaningful part of the intervention overall. The second-most-common ‘meaningful’ rating was given to ‘Reading health plan’, as reported by 50% (n = 42) of respondents (Table 3).

At 6 months, the proportion of participants fully agreeing that the intervention overall was meaningful had decreased to 33.3% (n = 27), and the proportion of participants fully disagreeing that the overall intervention was meaningful had increased from 3.6% (n = 3) at 3 months to 6.2% (n = 5). A dominant majority of 77.7% (n = 63) still reported that they found the intervention fully or partly meaningful, and 80.2% (n = 65) found the phone communication to be a meaningful part of the intervention. The proportion of respondents rating ‘Reading health plan’ as meaningful had decreased from 50 to 38.3% (n = 31), but it was still the content second-most often rated as meaningful after the phone communication. ‘Inviting significant others’ was rated as meaningful by the fewest respondents, only 6% (n = 5) after 3 months and 2.5% (n = 2) after 6 months (Table 3).

### Experiences of the intervention

In the qualitative analysis, three overarching categories of how the participants experienced the intervention were formed (Table 4). The first category, ‘Acknowledgment in a disconcerting situation’, and the second category, ‘Finding ways forward’, cover positive experiences of the intervention pertaining both to how participants perceived it and what meaning they attributed to the content and the mode of conduct in the eHealth setting. The third category, ‘Unmet expectations’, covers participants’ experiences of disappointment with and struggle to understand the intervention’s content and design.

**Table 4:** Overview of categories and subcategories

Category	1. Acknowledgment in a disconcerting situation	2. Finding ways forward	3. Unmet expectations
Subcategories	1.1 Feeling heard and respected 1.2 Remote support as a prerequisite and relief	2.1 Increasing awareness 2.2 Recognising strategies to act 2.3 Making and maintaining changes	3.1 Expecting disease-specific guidance 3.2 Unexpected content and barriers in the design 3.3 Efforts outweighing the rewards

### Acknowledgement in a disconcerting situation

Intertwined with how the participants experienced the intervention was the situation they were in at the beginning and throughout the intervention period. Several described an unsettling situation where they were tired, confused, worried about the future and questioned themselves. By perceiving that their experiences were important in the phone calls with the HCPs and through receiving support which did not add to their burdens, the participants described an overall sense of acknowledgment through the intervention. This is further elaborated in two subcategories: Feeling heard and respected and Remote support as a prerequisite and relief.

#### Feeling heard and respected

The participants described the phone calls as conveying to them the status of someone who matters. Their thoughts and experiences were requested by the HCPs and they felt listened to, like there was time for them to finish their sentences without being interrupted or having colliding agendas steering the conversation in a particular direction. One participant described feeling like there were no ulterior motives to the conversations and that their well-being, in the long run, was the number one priority. Perceiving that their words and experiences mattered to the HCPs, and that they could express themselves freely without feeling judged, was an important recognition of the legitimacy of their needs, emotions and reactions. Being heard and respected mattered in a situation where one could otherwise feel alone and vulnerable, especially if this was their first experience of mental illness.

Talking to someone, having an extra pair of eyes on you, to me that’s what you need, and especially if it’s the first time it happens, I felt almost lost, I didn’t know what happened, what kind of illness this is, I didn’t know if there was any kind of limit, so these phone calls with the nurse, that’s really what I appreciated the most. (Participant 2)

#### Remote support as a prerequisite and relief

Many participants considered the remote format of support to be essential. It enabled them to participate in the intervention and to access support without having to go to appointments in person, which relieved them of significant stressors. They felt like the limited energy they experienced as a consequence of their condition was being taken seriously as the remote format removed the need for them to use scarce time and energy on the planning and travel that physical appointments required. The possibility to write a message to the HCPs through the platform between scheduled calls made it easy for them to reach out when they needed to reschedule or wanted a professional’s point of view on something, and receiving a phone call while at work, home or wherever they were lessened the demands on them and made them feel cared for and looked after.

It felt somehow like it was on my terms even if it, you have a phone appointment to attend, that’s completely fine, I don’t need to physically go somewhere and travel and be somewhere on time or take time off work or anything like that, so it’s been really nice and very good for me. (Participant 9)

Many of the participants also described how talking to the HCPs by phone instead of face-to-face made it easier for them to talk freely and lower their guard. The phone worked as a kind of shield, both against the reactions of the conversational partner and against the uncomfortable situation of putting one’s emotions on display. Seeing reactions to their words in face-to-face interactions could make them feel awkward, lose track or censor themselves. The participants also described how remaining in their own milieu made them feel less like they needed to perform and like they could more easily allow the conversation to unfold from where they were in that moment of time.

I didn’t need to focus on you [the HCP], we didn’t even need to look at each other, which I also think contributed a lot to making it much, much easier to talk, it’s just someone listening. (Participant 8)

### Finding ways forward

The category ‘Finding ways forward’ covers participants’ experiences of the intervention as a support in a situation where their illness and the associated sick leave challenged them to reflect upon their lives, get an overview of their situation and to make decisions on how to act. This is further described in the subcategories: Increasing awareness, Recognising strategies to act and Making and maintaining changes.

#### Increasing awareness

Through the intervention, participants were provided with a forum in which to reflect upon how they were feeling and how they were progressing, something they might otherwise forget, avoid or struggle to find time to do. This reflection was encouraged in the phone calls but also in the self-rating and diary-like activities on the platform. The participants experienced that the intervention helped them untangle messy thoughts, get an overview of their situation or reflect more deeply on a particular concern. Their reflective processes were supported by the intervention HCPs, through their questions and reflections and through their mirroring of the participants’ words. The participants also described using the visualisation function of the self-ratings to keep track of how their symptoms evolved. They described how it could be difficult for them not to get carried away by their fluctuating feelings, and how seeing the progress they made allowed them to gain perspective on their feelings then and there. One participant described how she liked seeing the transformation in her graphs, starting at a low point at the beginning and moving up a notch as she started to feel better:

You made it up a notch or whatever you should call it, then I felt like you could see that in a graph. I like graphs; it suited me really well. And then maybe you went down again the next day but then at least you had… and then you could be reminded that, well, last week I was on the bottom of everything and now I’m not, so, you could, like, visualise that you were actually doing better. (Participant 4)

#### Recognising strategies to act

The participants described how they made use of their increased awareness through recognising actions they could take. They described how they could become aware of problems they needed to solve, imbalances in how they were prioritising their time and energy, ideas on how to organise life differently, or other directions they thought necessary to pursue as part of their recovery. When talking about such experiences, the participants mainly referred to discussions they had with HCPs during the phone calls. They described how they were encouraged to do most of the talking and how the HCPs followed up on their reflections by asking them to elaborate on their perspectives on a specific matter. They also described getting advice, information and encouragement on how to take action.

Somehow you realise, it felt like they helped you realise the questions that you had, they helped you realise the answers yourself. It wasn’t like ‘this is what we’ll do’ or ‘this is what I think you should do’ but… they helped you realise it yourself. (Participant 10)

#### Making and maintaining changes

The participants described how their situation required them to be active in a number of ways: active in getting the right treatment, active in making the workplace understand their situation, and active in changing their own attitudes and behaviours towards work or towards themselves. This was an ongoing process to most of them, something the intervention allowed them room to engage with. The participants described how the strategies laid out in the phone calls followed them into their everyday lives, and how an important aspect of the process was finding ways to maintain the changes, move forward and not to fall back into old habits.

I got the support I needed to move forward, otherwise you can get stuck on the same spot and, like, think you can’t manage and all that, but I felt like every time after every conversation I could take another step and it help me move forward, really. (Participant 7)

### Unmet expectations

This category covers the participants’ unmet expectations of the intervention, in terms of what kinds of support they felt were lacking and what kinds of support they felt were offered but did not appreciate. Some were uncertain about the exact purpose of the intervention, and this lack of clarity confused them as to why they should engage with it and what they could expect. The category is structured into three subcategories: Expecting disease-specific guidance, Unexpected content and barriers in the design and Efforts outweighing the rewards.

#### Expecting disease-specific guidance

Participants described expecting the intervention to give them access to experts on mental illness who could provide them with tools, personalised advice and general knowledge about their condition. Instead, they found that they were expected to do most of the talking during the phone calls, and they received little in return from the HCPs. They felt that it was up to them to find solutions to their problems and that the phone calls offered little more than a listening ear. They also described having other needs than ‘just talking about it’, especially if they already had people around who they felt they could talk to. Some described expecting information, but they felt that they were not encouraged to ask, neither in the phone calls nor through the platform, or if they did receive information, it offered little more than they already knew. Having expected to get tools and ideas to help them deal with their problems, they were disappointed with the lack of guidance on symptom management or advice on how to deal with their condition.

I guess I experienced a lot during the conversations that there was sort of this idea that I should put my condition into words, but sometimes I could ask for tips. I wanted more guidance, but it felt like the whole idea which I understood from their answers was more like that I should talk about how I was feeling. (Participant 11)

#### Unexpected content and barriers in the design

Whether or not the participants found the intervention meaningful overall, they primarily associated the intervention with the phone calls and regarded the platform as an appendage whose function in the intervention was not entirely clear. They expressed that, to achieve greater unity and clarity in the intervention, the platform would benefit from changes to both content and design. Many participants indicated that they would have preferred the platform to be designed in an app format rather than as a web page. The log-in procedure was described as cumbersome and as a barrier when one had very limited energy. Some also experienced technical problems with logging in to the platform, and one participant never managed to access it. Issues with the design also pertained to particular functions of the platform. Participants wanted to use the self-rating function to track their progress, but because graphs were visualised weekly, tracking progress in a longer perspective was difficult.

And then I don’t think you could see it over a longer time period either, so this diagram could have been good or like what I figured was the primary purpose with it to, like, follow a curve where you could see that [in] the last couple of months your condition slowly got better and better, for example, but you couldn’t really do that or the diagram didn’t function that way; you couldn’t use it like I think it was intended. (Participant 5)

Some participants indicated that some aspects of the intervention were not exactly clear to them. They were unsure of what they were supposed to do and accomplish in the phone calls and on the platform, and what kind of personal gain they could expect from the intervention. For example, one participant described how she did not understand the logic behind the planning of the phone calls, which appeared to her as detached from her current needs and situation, making her question whether the planning was really intended to follow her needs or if there was another, hidden, structure:

In the beginning it was like every week and then all of a sudden it was thinned out. I think it was those kinds of things that made it feel detached because it wasn’t that anchored; I mean I was still feeling really bad. (Participant 11)

In hindsight, participants expressed that if they had understood more about the logic behind the different activities proposed in the intervention, perhaps they would have given them more of an effort. However, many felt unsure about inviting their extended network to their platform, and very few chose to do so. Either they described not seeing what good it could do, or they felt it would compromise their integrity, and they valued having a private forum in which to express their thoughts and feelings. Those who already had good support from family and workplace representatives and were communicating well with them, saw little benefit in inviting them to the platform. Equally, if they described lacking a supportive network, this also served as a barrier to inviting others as they either did not know whom to invite or did not feel safe letting people know how they were really doing.

#### Efforts outweighing the rewards

Some participants experienced that the intervention required more of them than it gave in return. Needing to prioritise their efforts towards what gave the greatest reward made them shy away from using parts of the intervention, or from engaging at all, when they felt that the efforts outweighed the rewards. For example, they felt discouraged from using the platform when they received so little feedback on those activities from the HCPs in the phone calls. One participant described how there was a kind of tunnel vision throughout the intervention, where so much focused on the phone calls, and that the use of the platform was forgotten by both the staff and the participant. Not receiving feedback on their ratings or other activities made some participants feel like there was no point in continuing to use the platform. Some participants also described an imbalance between their stake and the return in the phone calls with the HCPs. Talking to yet another person about their situation could be tiring, and they did not want to have to go through difficult subjects once again. Finding the time and energy to engage in yet another activity was also a source of stress for some of the participants, and they described how obstacles such as difficulty finding time for phone calls during working hours and feeling like they should take the chance to use the platform properly added to their stress.

Despite being on a level where you can barely, like I said, barely manage to wash your hair, there’s also this kind of need to be good. I have this good girl mentality which made me feel like now that I’ve been chosen, I got the chance to be a part of this, I felt, like, this pressure on myself to log in and use the platform. (Participant 8)

#### Interpretative synthesis: a lowered threshold to partnerships - support within reach, when needed

An interpretative synthesis of the quantitative and qualitative findings resulted in the overarching theme of meaningfulness as constituted by perceiving a partnership, characterised by the sensation of having the support of a professional within reach, when needed. While physical appointments in regular care required a lot from the participants, the remote format of the intervention was considered suitable to their needs, as it lowered the threshold to receiving professional support. Support within reach also covers the relief of evading face-to-face appointments without compromising the sense of being cared for and looked after by empathetic and competent professionals, mainly through the connection established and maintained in the phone calls. In the descriptive statistics, no other activity came near the meaningfulness of the phone calls with HCPs. This activity was regarded as meaningful by more than 80% of intervention participants at both follow-ups, and it was the activity reported as most meaningful when participants were asked to choose. Furthermore, the possibility of contacting HCPs through the platform and by phone, between scheduled calls, was also reported as meaningful by 20–30% of intervention participants, further strengthening the interpretation of ‘within reach, when needed’ as a central mechanism in the intervention.

However, it appears that easy access to HCPs only covers part of the experience of meaningfulness. If participants did not perceive the support as relevant in response to their particular expectations and needs, then access did not matter. We found that a key to meaningfulness was thus the establishment of partnerships, which both acknowledged the patient’s situation and involved the HCPs’ guidance and encouragement to find ways forward. Achieving partnerships which recognised the participants’ needs and met their expectations could thus be interpreted as central to the experience of meaningful support. Conversely, a failure to achieve proper recognition of and response to the patient’s needs could be understood as blocking meaningfulness.

## Discussion

This mixed methods analysis used questionnaires and interview data to explore participants’ experiences of a person-centred eHealth intervention. It is important to understand what constitutes meaningfulness for patients with CMDs, both in regard to recovery [20, 21] and when designing health care support, also in order to avoid engaging patients in activities which add to their stress or are perceived as a burden. The results of the present study indicate that for a majority of the participants, the person-centred intervention was perceived as meaningful. In order to be a meaningful support, it is important to clarify expectations and needs, and to ensure that purposes of proposed activities are transparent and appreciated.

The identification of intervention processes recognised by participants as meaningful to their experience of the intervention, and beyond, in their recovery and sick leave process can contribute to understanding how PCC interventions work, or why they do not work as intended. The integration of data strands resulted in an overarching interpretation of the intervention’s meaningfulness as constituted by perceiving a partnership, characterised by the feeling of having the support of a professional within reach, when needed, through the eHealth format. Furthermore, we conclude that the most meaningful processes of the intervention took place in the phone calls, in which positive experiences of acknowledgment and reflective, change-oriented processes could occur. In accordance with literature describing partnerships in PCC, this could be understood as another testament to the importance of establishing an emotionally supportive, trusting relationship building on a common understanding [15, 19, 38]. Moreover, the findings of the study add to prior evidence that such relational qualities are not only possible in remote settings but are sometimes even facilitated outside of the traditional health care environment [39]. Our findings are also congruent with prior research suggesting that personalisation and access to professional support are valued features of eHealth interventions for CMDs [27–29].

However, despite the intention to co-create support according to each participant’s experiences and needs, some participants indicated that the intervention did not correspond to their expectations or met their needs. A recent study evaluating a related intervention among people diagnosed with chronic conditions highlighted similar challenges with unmet expectations due to unclarity of roles and unspoken expectations about what each partner can contribute to the partnership [34]. This suggests that in order for PCC interventions to be experienced as meaningful, it is important to make sure that patients understand why and how the intervention work and what they are intended to achieve, and to openly communicate expectations of both patient and HCPs to limit the risk of failing to meet needs because they are unrecognised. Additionally, it is worth considering that even if needs are recognised, they can be in collision with the intervention’s agenda or challenging to combine with the ethical principles of PCC. For example, if patients express a need to be taken care of, or that they are ill equipped to manage their illness, recognising their needs while also encouraging them to be active partners in care is a delicate task for the HCP. When the intention is to support capabilities and recognise needs, it is important to find a balance where patients are neither abandoned to self-management nor stripped of their agency [40].

Among the participants in this study, where the majority where on sick leave due to stress-related conditions, many participants described how meaningful and important it was to them that the HCPs encouraged and supported them in the importance to take a step back, which can be understood as a reminder of vulnerability and needs. Further, self-stigma is common among patients with mental illnesses, and self-stigmatising thoughts can be reinforced by having difficulties managing everyday life and work [41, 42]. This was evident also in our material, expressed as a form of vulnerability where the HCPs’ expressions of recognising and taking the illness seriously were valued and important forms of acknowledgement. To patients with CMDs, a person-centred approach may thus aid in avoiding to push their recovery too fast or too hard.

Furthermore, in concluding that the most meaningful part of the intervention was the access to and provision of HCP support through the phone calls, it was also evident that the platform failed to assert any greater meaning as support to the intervention participants. Our interpretation is that the platform had potential to support reflective and recovery-oriented processes, but in its current form, the purpose was not sufficiently clear to the patients, and perhaps not to the HCPs either considering the lack of attention given to platform activities in the phone calls. As studies on acceptability and engagement in eHealth interventions clearly suggest that tailored content and feedback strengthen participants’ engagement [28, 43, 44], this could be important to address as an area of improvement.

An unexpected finding was the lack of willingness to invite family and professional contacts to the platform. The reasons given in the interviews highlight the value participants placed on having a private space in which to express themselves in the turbulent process of illness and sick leave, or that the participants felt that they already had adequate communication with others. The need to invite, inform and share information with an extended network was indeed smaller than anticipated from previous studies on the importance of support in RTW processes [45–47]. However, this finding is congruent with a study evaluating a person-centred eHealth support targeting people diagnosed with chronic conditions [34], which could suggest the need to seek new ways to include other kinds of support in the PCC process.

### Strengths and limitations

A strength of the present study is the purposive sampling of intervention participants with both positive and negative experiences of the intervention, which was representative of the full intervention group (Table 2). In interviews, it can be difficult for informants to express unpleasant or negative views, especially if this can be read as critique. During the interviews, measures were taken to express the value of both positive and negative experiences of the intervention in relation to the aims of a process evaluation, and the interview data were considered rich, complex and full of nuances. The first author, who conducted the interviews, had no prior communication with the informants and had no significant role in delivering the intervention, which hampers the risk of social-desirability bias.

Using a concurrent mixed methods approach enabled the analysis to accommodate experiences at the group and individual levels, and to triangulate the different findings [32]. A further strength is the collaborative process in which the analysis was performed, while the construction of an intervention-specific questionnaire for the quantitative strand can be considered a limitation. While this was considered a feasible way to capture the processes and activities of this specific intervention, it impedes comparisons of experiences between different interventions.

Another limitation of the study is that meaningfulness has not been conceptualised as meaningful in relation to a certain outcome, but as a personal judgment of every participant’s own experience of the intervention [20]. However, it is precisely the individual attribution of what is meaningful to each and every one that constitutes the concept’s relevance in person-centred care. Furthermore, exploring participants’ experiences can help to guide the understanding of what happens in an intervention and capture unexpected pathways [25]. This is considered particularly relevant in understanding what enables or blocks perceiving an intervention as supportive, considering the experiential dimension of support.

## Conclusion

The majority of participants reported that the person-centred eHealth intervention was meaningful to use. Participants’ experiences of meaningfulness of the intervention were constituted by a lowered threshold to forming care partnerships, in which support was within reach, when needed. However, if the content of the intervention was not in accordance with individuals’ needs or expectations, access alone did not suffice to constitute meaningfulness. The most meaningful processes and activities in the intervention occurred in the phone calls between patients and the intervention HCPs, and the platform failed to assert any greater supportive influence due to unclarities in the content and barriers in the design. If pitfalls in the design are addressed, the format of the intervention and the person-centred approach underpinning its content and design have potential to function as a valued and anticipated support for patients with CMDs.

### Electronic supplementary material

Below is the link to the electronic supplementary material.

Supplementary Material 1

## Data Availability

To maintain participant confidentiality, per information provided in the informed consent, study data is not publicly available. Reasonable requests to the corresponding author will be considered and a confidentiality assessment will be performed at each individual request.

## Abbreviations

CBT: Cognitive behavioural therapy
CMD: Common Mental Disorder
GPCC: Gothenburg Centre for Person-Centred Care
HCP: Health Care Professional
MRC: Medical Research Council
PCC: Person-Centred Care
RCT: Randomised Controlled Trial
