# Supplementary material for: A lowered threshold to partnerships: a mixed methods process evaluation of participants’ experiences of a person-centred eHealth intervention
Source: BMC Health Serv Res. 2023 Nov 2;23:1193. doi: 10.1186/s12913-023-10190-7 (PMC10621160; doi:10.1186/s12913-023-10190-7)
Supplement: Supplementary file 1 — Supplementary Material 1 [file 12913_2023_10190_MOESM1_ESM.docx]

**Interview Guide**

**Question Area 1 - General about the Support:**

When you gained access to this support, how were you feeling and what were your thoughts about the support offered by the study as a whole?

How did it meet your expectations and needs?

What has this support meant to you?

**Question Area 2 - About the phone conversations:**

- How did you experience the phone conversations with the study staff?
- How did you perceive the contact with the study staff you had conversations with?
- Overall, what would you say the conversations were about?
- What significance did the conversations hold for you?
- Did you feel anything was lacking in or during the conversations?
- If you compare these conversations with other contacts you've had within healthcare regarding the condition you were on sick leave for, do you find them similar or different? If different, how so?

**Question Area 3 - About the Platform:**

How did you experience the MyHealth platform?

- How did you find the platform's various functions (accessing links to information about the condition, inviting relatives, assessing your daily condition, reading/writing health plans, sending messages to staff)?
- How did you use the platform?
- What did the platform mean to you?
- Are there any functions or features in the platform that you feel are missing?

**Question Area 4 - About Person-Centred Care:**

Person-centred care involves working towards a partnership between the patient and the healthcare provider, as well as the patient's network. It emphasizes shared expertise, where the patient's understanding of themselves and their life is as important as the caregiver's knowledge. It's also about ensuring that the contact is not governed by the healthcare system's agenda, but that the patient has space to express how their life is affected and what thoughts, needs, and desires they have regarding their condition. It starts from a perspective that everyone, even when ill, has abilities that contribute to their well-being.

Did you feel this in the intervention?

If yes, how so?

If not, can you provide examples of where it was lacking?

**Question Area 5 - General Well-being:**

How do you feel now compared to how you felt when you joined the study?

What has had the most significant impact on your overall well-being?

Is there anything else you would like to add?
